# Artificial intelligence-produced radiographic enhancements in dental clinical care: provider and patient perspectives

**DOI:** 10.3389/froh.2025.1473877

**Published:** 2025-02-14

**Authors:** Lyubov D. Slashcheva, Kelly Schroeder, Lisa J. Heaton, Hannah J. Cheung, Brenda Prosa, Nicole Ferrian, Jesse Grantz, Deborah Jacobi, John J. O’Malley, Michael Helgeson, Eric P. Tranby

**Affiliations:** ^1^ Apple Tree Dental, Corporate Office, Brighton, MN, United States; ^2^ Apple Tree Dental, Fergus Falls Center, Fergus Falls, MN, United States; ^3^ Analytics and Data Insights, CareQuest Institute for Oral Health, Boston, MA, United States; ^4^ Apple Tree Dental, Fairmont Center, Fairmont, MN, United States

**Keywords:** artificial intelligence, dental caries, periodontal disease, software, dental providers, dental patients

## Abstract

**Introduction:**

Artificial intelligence (AI)-based software can be used with dental radiographs to facilitate dental providers’ diagnoses and to educate patients about their oral health conditions. The goal of this study was to survey dental providers and patients about the use of AI-enhanced radiographs in the diagnostic and patient education processes.

**Methods:**

Within their Community Collaborative Practice model, Apple Tree Dental in Minnesota implemented the use of an AI software platform that annotates carious lesions and periodontal measurements on dental radiographs. Before and after implementation of this software, providers (dentists, dental hygienists, dental therapists, and dental assistants) were surveyed about what benefits and challenges they anticipated and experienced in using the AI software. A small-scale study of patients who viewed AI-generated annotations on their own radiographs examined patient perspectives on the use of this software.

**Results:**

Dental therapists reported using the software most often, with 57.2% using the software at least 50% of their clinical time; 79% of dental assistants reported using the software 25% of the time or less. While the majority of providers (*n* = 70 for Survey I; *n* = 53 for Survey II) said that AI enhancements would help facilitate patient education efforts, providers’ confidence in the ability of the AI software to improve diagnosis of dental caries and periodontal disease and its ability to improve the efficiency of their work was mixed. Patients (*n* = 25) found reviewing the AI-produced visual aids used by their dental provider to be helpful in understanding their oral health, and a large proportion (92%) said they planned to follow through on recommended treatment.

**Discussion:**

While provider and patient perceptions of the use of AI software in dental care were positive overall, attitudes among providers were mixed regarding its effectiveness in diagnosing dental disease and improving work efficiency. More research is needed to determine whether use of AI software in clinical dental practice produces changes in treatment recommendations by providers or in patient adherence to these recommendations.

## Introduction

### Use of and advancements in dental radiograph technology

Radiographs were first used to take dental images in 1896, although they were not introduced into widespread use in dentistry until the 1950 s (1). Since then, dental radiographs have become “the cornerstone of many patients’ dental voyage, from diagnosis to treatment planning, to conducting and re-evaluating therapies” [(2); page 1]. These two-dimensional images can be used to detect issues such as loss of tooth structure (e.g., as a result of carious lesions), the presence and extent of a periapical infection, and loss of bone surrounding a tooth (3). In a review of over 11,000 dental patient records, intraoral periapical radiographs were used primarily to diagnose caries (67%), periodontal disease (16%), and dental trauma [7%; (4)].

Accurate interpretation of dental radiographs has historically relied on both the clinical experience of the dental providers reading and analyzing the images and the quality of the images themselves. A systematic review of factors that influence errors in the interpretation of dental radiographs identified several provider- and situation-related elements: the clinical experience, knowledge, technical ability, training, and cognitive load of the provider, as well as the time pressure under which the images are interpreted and complexity of the presenting case (5). Dentists report the dental conditions most prone to interpretive errors include dental caries, cracked teeth, and cervical resorption of teeth; they also attribute interpretation errors to lack of provider clinical experience and workload as well as poor image quality (6). Reviews of panoramic radiographs suggest that nearly 90% of images have at least one positioning or preparation errors, including the patient's tongue not being placed on the palate, the patient's chin tipped too high or low, and exposure errors (7, 8).

Dental radiographs are key in the paradigm shift away from surgical treatment of dental disease to minimally invasive treatment and disease monitoring. Dental caries, historically treated through removal of carious lesions and healthy tooth structure and the placement of restorative material, may now be prevented and arrested by minimally invasive treatments such as fluoride varnish, silver diamine fluoride, sealants, atraumatic restoration treatment, and others (9). In a small pilot study, minimally invasive non-surgical periodontal therapy (MINST) was found to be comparable to conventional non-surgical periodontal therapy (CNST) on periodontal outcomes such as probing depth and bleeding on probing; further, MINST performed better than conventional therapy with regard to patient comfort and gingival recession (10). Sealing the dental pulp in adult teeth after pulpotomy with materials such as bioactive hydrophilic calcium silicate cements is showing promise as an alternative to traditional endodontic therapy (11). Minimally invasive treatments are particularly welcome as a way to treat patients with dental care-related fear and anxiety by avoiding invasive, anxiety-provoking procedures involving intraoral injections and handpieces (12). Effective use of minimally invasive treatments relies on accurately identifying, treatment planning, and then monitoring the arrest or progression of disease, often via dental radiographs.

Artificial intelligence (AI) annotation of dental radiographs has the potential to improve the consistency of disease identification, diagnosis, treatment planning, and monitoring. Systems using AI are able to analyze radiographs, dental records, and intraoral images to assist providers in developing personalized treatment recommendations for their patients (13). AI can enhance radiographs in order to improve providers’ ability to visualize oral structures, improving disease diagnosis and monitoring (14). AI can be used to detect a multitude of dental conditions, such as caries, periapical infections, bone loss due to periodontal disease, and others (15). Machine learning algorithms—sets of rules used to discover patterns and insights in large datasets (16)—are being used to enhance and analyze dental radiographs in service of dental disease detection, among other uses (17). For example, researchers assessed a deep learning algorithm (a type of machine learning) in diagnosing periapical radiolucencies on panoramic radiographs; this algorithm outperformed 14 of 24 oral and maxillofacial surgeons in its diagnoses (18). Within deep learning, deep convolutional neural networks (CNNs) are able to detect dental caries and periapical periodontitis lesions with precision, although they currently perform better when detecting severe, rather than smaller lesions (19). Further, CNNs are able to process several images quickly, allowing for the detection of more tooth anomalies, such as carious lesions, in a shorter amount of time (20, 21).

In addition to improving and facilitating interpretation of radiographs for dental providers, AI can be used as a tool to educate patients about their oral condition, particularly in the use of minimally invasive treatment options. Dental patients rely on dental providers for accurate interpretation of radiographs and recommendations for subsequent treatment (22). Providers are able to use visual output generated by AI and machine learning to better help patients visualize and understand their dental conditions, which may motivate patients to adopt more effective oral health habits and to seek treatment earlier than they would without the visual output (23). When presented with radiographs with an AI-generated colored overlay that highlighted carious lesions, patients were better able to identify these lesions than when they were shown radiographic lesions pointed out with an arrow (24). When surveyed, patients are generally supportive of the use of AI in dentistry, and anticipate more confidence in diagnoses, reduced time for treatment, and more personalized, evidence-based disease management with use of AI in dentistry (24, 25).

### The use of AI technology in a dental organization

Apple Tree Dental was founded as a non-profit dental organization in 1985 in the Minneapolis/St. Paul area of Minnesota in the US. Their mission is “to overcome barriers to oral health” through a Community Collaborative Practice model, which is characterized by collaboration with community partners to deliver oral health services where people live, go to school, or receive other health and social services (26). At the organization's earliest phase, they delivered on-site dental care to residents in long-term care facilities. They have since expanded their services to deliver dental care to patients of all ages and abilities through nine Centers for Dental Health in partnership with over two hundred community sites. Community Collaborative Practice encourages oral health professionals (i.e., dentists, dental hygienists, dental therapists, and dental assistants) to practice at the top of their license with support from non-clinical staff and leadership. There are about three hundred staff members employed across the nine Centers in Minnesota. A key component of this practice model engages partnerships with local leaders, health and social service providers, funders, researchers, and other stakeholders that share in Apple Tree's vision “to inspire partnerships that foster healthy communities.” With over 85% of their patients insured through public programs and the cost of providing care exceeding public program reimbursement, innovative and efficient solutions are essential to Apple Tree's continued success.

Apple Tree's Learning Health System uses their Community Collaborative Practice model to prioritize innovation, research, and improvements in the oral health delivery system. Infrastructure enabling innovation, research, and the delivery system started early on at Apple Tree. Recognizing the potential value of the data contained in dental records in the mid-80's when dental records were largely concise paper charts, Apple Tree created their own electronic systems to capture and use data, which included using primary dental finding codes, billing and scheduling functions, and tracking communication of treatment recommendations to patients and/or their responsible party. At present, Apple Tree's longitudinal database contains records for over 206,000 patients across all ages of documented visits (1.6 million) and dental procedures (5.8 million). The breadth of their documentation on medical conditions, dental conditions, and dental care provided over the years offers a valuable resource for understanding the connection between oral and systemic conditions. Through Apple Tree's Innovation Teams, they have been able to recognize the added value of investing in technology and software to grow the organization's data analytic capacity.

In recent years, Apple Tree adopted AI software (OverJet AI technology) in its clinics. This AI software is applied to radiographs to identify and highlight potential dental pathologies such as bone loss, density changes in tooth structure, and other tooth anomalies (see Appendix A). Implementation of this software assists clinicians in their diagnostic capabilities, encourages early detection and minimally invasive treatment of oral disease, and is a tool for patient education about their oral condition.

### Programmatic elements in the implementation of clinical AI software

Apple Tree's Innovation Teams include Clinical Innovations and Research. These Teams, consisting of executive leaders and clinical staff, were central to exploring options for integration of AI tools into clinical care. Once radiograph AI annotation became a focus and priority for both Teams, connection to a specific software company was established. A technology innovation grant was secured to support the first year of subscription for this software.

To support clinical staff receptiveness toward adopting AI software into their clinical routines, the Chief Executive Officer created a brief video that was circulated to all staff. This video explained the industry trends and opportunities for innovation relative to AI and described the grant-funded implementation of the specific radiograph annotation software that was being launched. A baseline survey was conducted to assess staff perceptions of the AI software and their anticipated experiences with the radiograph annotation tool in relation to their workflows and diagnostic accuracy and efficiency.

The innovation was launched first at one of the nine Centers for Dental as a pilot site. Training for this Center was conducted in real time through a live webinar. A champion provider at this Center created a resource document for review by peers describing how and why they have used the new software with tips on integrating the tool into existing workflows. The remaining Centers received training and onboarding on the software through an asynchronous webinar format with a live organization-wide question and answer session.

Utilization reports were sent from the AI software company on a routine basis to track the extent to which providers at specific Centers utilized the software. Regular communication with the AI software company staff aided technical assistance to optimize staff uptake and experience with using the software. Several advancements and new features were released during the implementation of the software and were communicated to staff as updates and points of encouragement for use of the software. These updates included clearance from the Food and Drug Administration (FDA) for additional patient populations (children), additional annotations of specific radiographic anatomy, and a dashboard that reported measures of utilization and treatment acceptance. Project leaders from Apple Tree participated in calls as beta-testers for prospective and new software features.

Clinical staff members completed evaluation surveys to assess their perceptions, utilization, and experience with the AI software. Feedback from the first survey was utilized to identify additional barriers to staff uptake of the software (e.g., to improve the workflow barriers, a button was added to the patient record to directly open radiographs for each specific patient in the software). A small-scale patient survey was also conducted to assess the value of the software for patient education. A grant report was created to provide an annual summary of AI software implementation. Apple Tree leadership met with AI software staff to discuss options for continued software use, such as continuing a standalone software subscription and/or adding an AI overlay into the radiograph software previously and currently used by the organization. The goal of this study is to examine oral health providers’ and patients’ perspectives toward the use of AI software in clinical dental care.

## Methods

Provider and patient surveys occurred during two stages, immediately following Overjet training and approximately four months after implementation (a third survey will be administered approximately 12 months after implementation of the AI software; see Appendix B for the dental provider survey). As all the providers are employees of the same organization (Apple Tree Dental), to maintain respondents’ privacy, no demographic data were collected from providers as part of the data collection process beyond provider type. A patient survey was administered approximately nine months after the AI software was implemented by one dental provider after this provider educated patients about their oral health status using the AI software to annotate radiographic findings. After reviewing radiographs with AI annotation with patients to explain their oral condition and discuss treatment recommendations, the provider gave these patients a printed (paper) survey while the patients were in the operatory (see Appendix C for the patient survey) and guided patients through questions about their dental care and perception of the AI software. Patients completed the demographic questions independently. All surveys included an open-ended response section for providers and patients to write in additional perceptions about their AI software experience. Both provider and patient surveys were reviewed and determined to be exempt by WCG IRB.

## Results

### Provider survey

Survey I had responses from 70 out of 192 clinical team members, including dentists (*n* = 10), dental hygienists (*n* = 19), dental therapists (*n* = 12), and dental assistants (*n* = 29). Survey II had responses from 53 out of 192 clinical team members including dentists (*n* = 18), dental hygienists (*n* = 7), dental therapists (*n* = 7), and dental assistants (*n* = 21).

**Figure 1:**
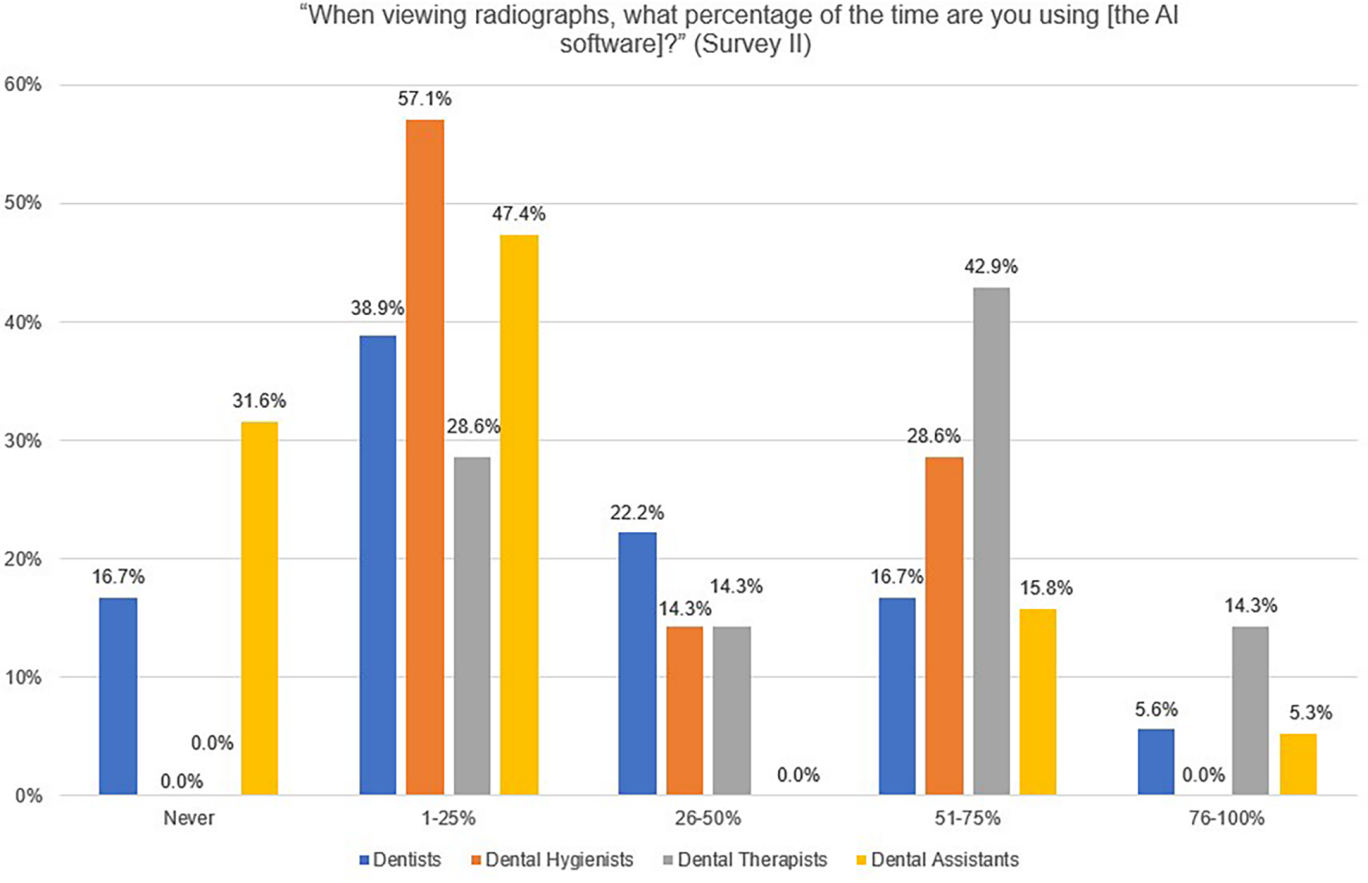
Providers’ responses to the Survey II question, “When viewing radiographs, what percentage of the time are you using [the AI software]?”.

Providers were asked in Survey II (after implementation of the software), “When viewing radiographs, what percentage of the time are you using [the AI software]?” Dental therapists reported using the AI software most often, with 14.3% using it 76%–100% of the time and 42.9% using it 51%–75% of the time (Figure 1). Dental assistants reported using the software the least, with 79% reporting using the software 25% of the time or less.

**Figure 2:**
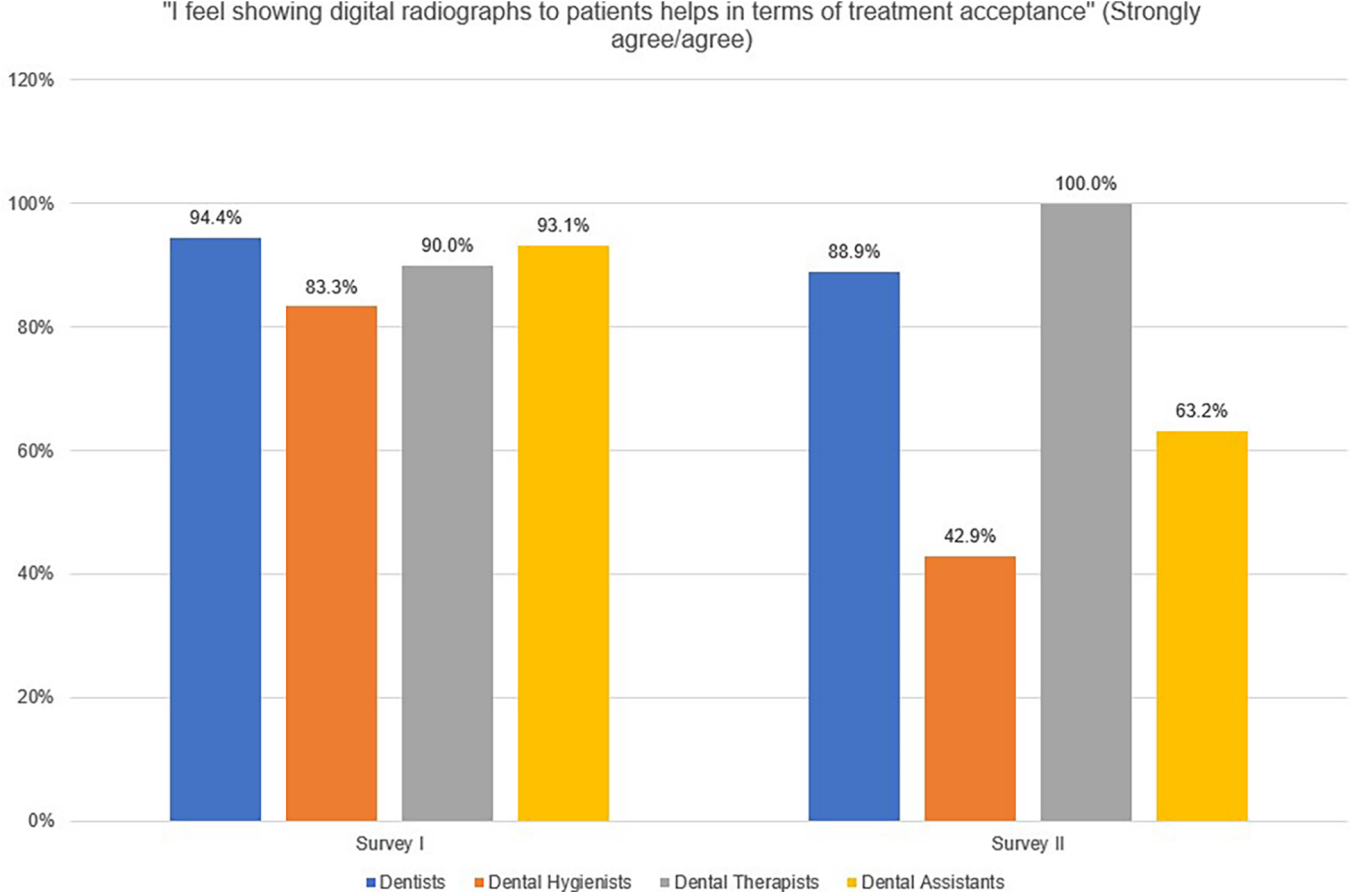
Providers’ responses (strongly agree/agree) to the statement, “I feel showing digital radiographs to patients helps in terms of treatment acceptance”.

When asked, “I feel showing digital radiographs to patients helps in terms of treatment acceptance” in Survey I, a high proportion of dentists (94.4%), dental hygienists (83.3%), dental therapists (90.0%), and dental assistants (93.1%) responded, “strongly agree” and “agree” (Figure 2). In Survey II, a lower proportion of dentists (88.9%), dental hygienists (42.9%), and dental assistants (63.2%) responded that they “strongly agree” or “agree” to this statement, while all dental therapists strongly agreed or agreed with this statement (100%).

Provider responses to the question, “How confident are you that radiographs help accurately identify caries?” for Survey I remained consisted with a large proportion all providers (dentists, 94.7%; dental hygienists, 91.6%; dental therapists, 100.0%; and dental assistants, 93.1%) responding “completely confident” or “fairly confident”. In Survey II, the proportion of dental hygienists (100.0%) and dental therapists (100%) who responded “completely confident” or “fairly confident” increased while the proportion of dentists (66.7%) and dental assistants (79.0%) who responded “completely confident” or “fairly confident” decreased.

When providers were asked, “How confident are you that radiographs help accurately identify periodontal disease?”, the proportion of dental therapists who reported being “completely confident” or “fairly confident” in radiographs helping to accurately identify periodontal disease remained about the same for Survey I (100%) compared with Survey II (100%). The proportion of dentists who reported “completely confident” or “fairly confident” decreased from Survey I (89.5%) to Survey II (55.6%). A larger proportion of dental hygienists responded “completely confident” or “fairly confident” in survey II (100%) compared with survey I (75.0%). The proportion of dental assistants who responded “completely confident” or “fairly confident” also increased in survey II (73.7%) compared with survey I (62.0%).

**Figure 3:**
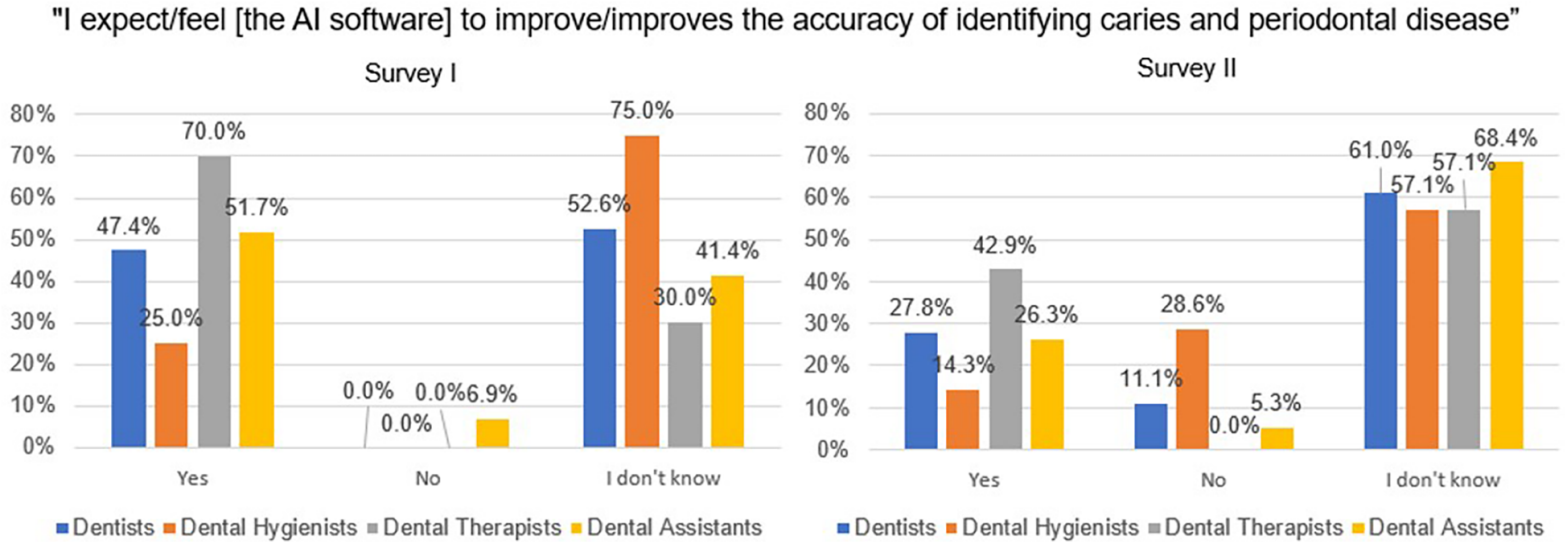
Providers’ responses to the statement, “I expect/feel [the AI software] to improve/improves the accuracy of identifying caries and periodontal disease” before and after using the AI software.

Since providers had not used the AI software before completing Survey I, the survey asked the following question, “I expect [the AI software] to improve the accuracy of identifying caries and periodontal disease” in the future tense (Figure 3). Responses from providers included dental therapists (70.0%) reporting the highest proportion of confidence with their response “yes” compared with dentists (47.4%), dental hygienists (25.0%), and dental assistants (51.7%) who responded “yes.” The question was asked again in survey II, although in the present tense, “I feel [the AI software] improves the accuracy of identifying caries and periodontal disease”. To this variation in the question after using the AI software, the proportion of dentists (27.8%), dental hygienists (14.3%), dental therapists (42.9%), and dental assistants (26.3%), who responded “yes” decreased for all providers.

**Figure 4:**
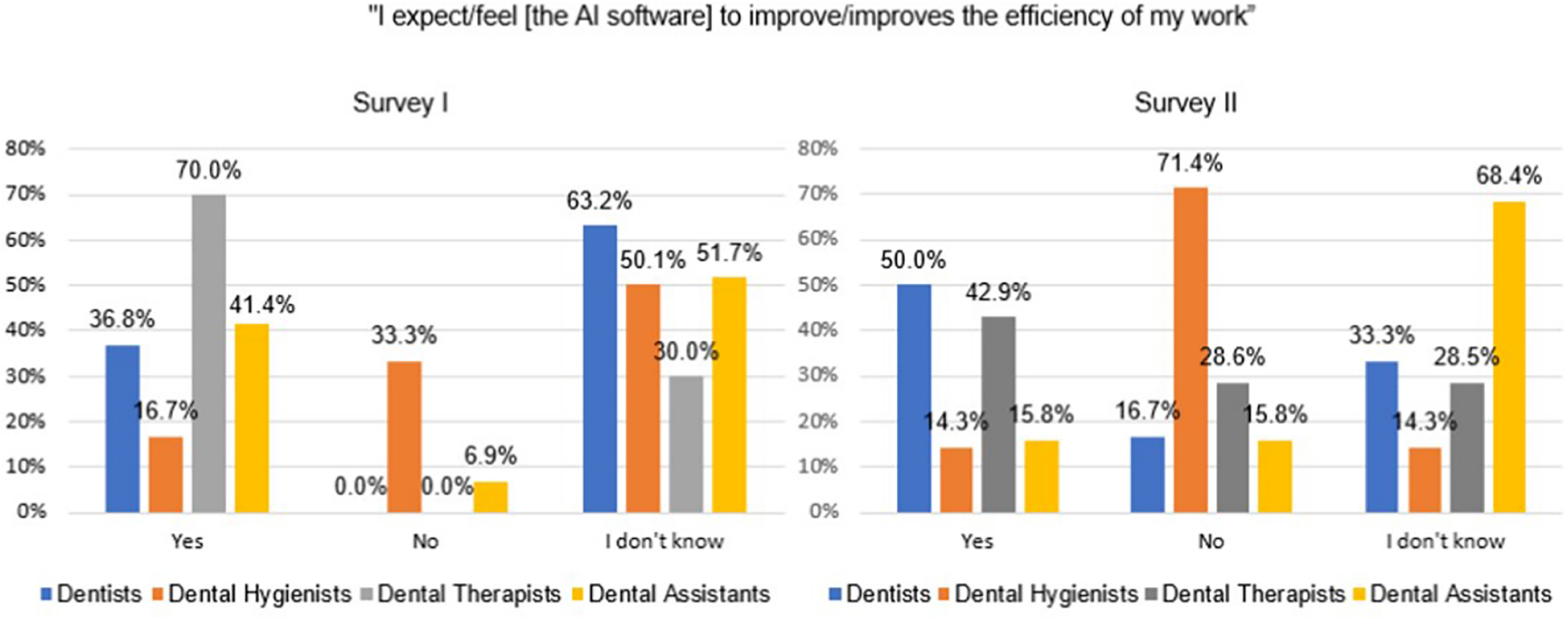
Providers’ responses to the statement, “I expect/feel [the AI software] to improve/improves the efficiency of my work” before and after using the AI software.

The largest increase of providers responding “yes” to the statement, “I expect [the AI software] to improve the efficiency of my daily work” between Surveys I and II was in dentists’ responses (13.2%) compared with a decrease in “yes” responses from dental hygienists (2.4%), dental therapists (27.1%), and dental assistants (25.6%; Figure 4). Even after using the software, dental assistants (68.4%) reported most often that they “don't know” if the software would improve their efficiency followed by dentists (33.3%), dental hygienists (14.3%), and dental therapists (28.5%). The largest proportion of providers who responded “no” after using the software were dental hygienists (71.4%) followed by dental therapists (28.6%), dentists (16.7%), and dental assistants (15.8%).

**Figure 5:**
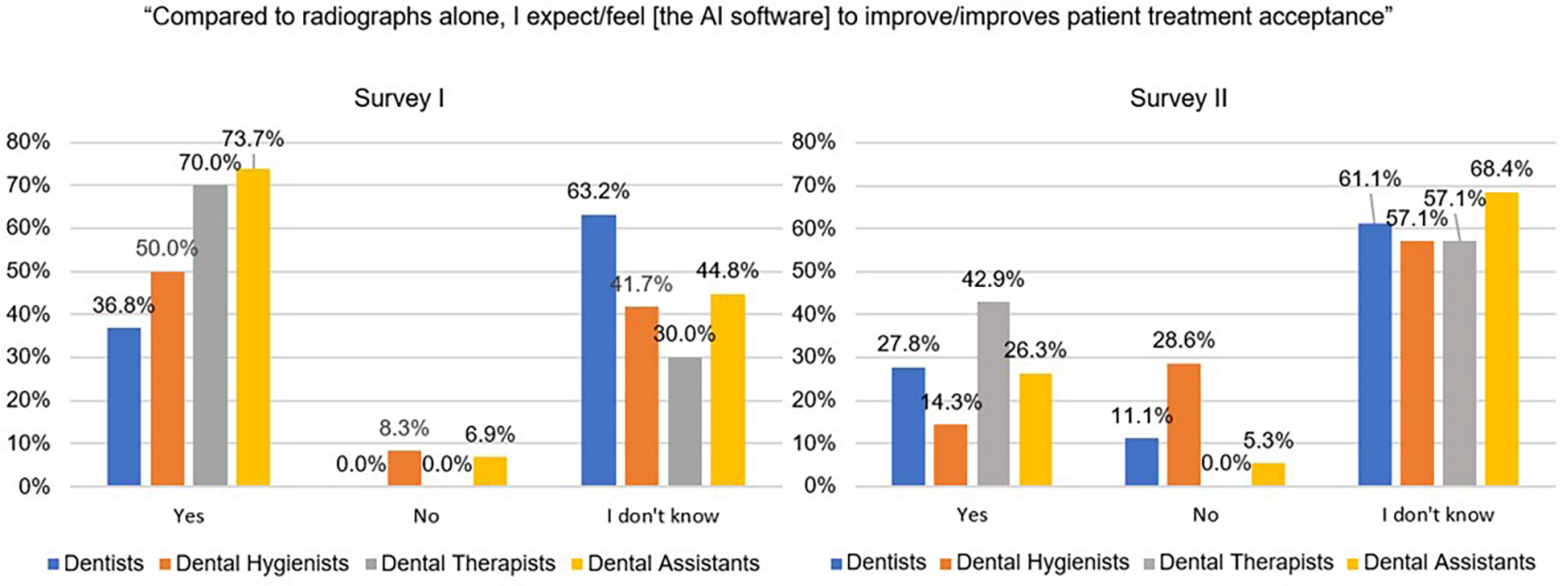
Providers’ responses to the statement, “Compared to radiographs alone, I expect/feel [the AI software] to improve/improves patient treatment acceptance” before and after using the AI software.

A higher proportion of dental assistants (73.7%), dental therapists (70.0%), and dental hygienists (50.0%) responded “yes” in Survey I when asked “Compared to radiographs alone, I expect [the AI software] to improve patient treatment acceptance,” compared with “yes” responses from dentists (36.8%; Figure 5). In survey II, when asked the same question, a larger proportion of dental therapists (42.9%) responded “yes” compared with dentists (27.8%), dental assistants (26.3%), and dental hygienists (14.3%).

### Patient survey results

Patients (*n* = 25; see Appendix C for the patient survey and Appendix E for patient demographics) completed a survey after receiving education about their oral condition using the AI software. The software was used to highlight anomalies on the patient's x-rays that were concerning to the dental provider and needed further attention with a follow-up appointment. Two different providers administered the patient surveys, and all patients who were approached to complete the survey agreed to do so.

Most patient survey respondents (76%) reported their dental provider had gone over the x-rays with the patient in the past to show carious lesions and bone loss. After seeing x-rays with the AI software highlighting, all study participants reported they thought the addition of the software highlights were very helpful (80%), helpful (16%), or slightly helpful (4%). A large proportion of participants responded that they plan to follow up with treatment recommendations after viewing the AI software annotations (92%).

### Qualitative results—provider survey write-in responses

Many survey write-in responses were related to providers being skeptical and cautious about using an AI platform to recognize oral disease (see Appendix D). Although there was some apprehension expressed about using new technology, there was also a general sense of interest, curiosity, and flexible thinking towards the AI software. A difference was noted from Survey I compared with Survey II with providers expressing sentiments of worry about relying on the AI software to diagnosis radiographic anomalies in Survey I compared to the second survey where providers expressed understanding that the AI software is a tool to identify dental disease and provide patient education.

A difference was also seen among provider mindsets with some providers expressing challenges with the AI software logistics and frequent login requirements, while other providers managed to incorporate the logistic requirements into their routines and found the use of the AI software as an overall time saver when examining radiographs, diagnosing oral disease, and creating treatment plans.

“Most of the time it makes exams faster.”—Dental assistant

“[The AI software] lines up all the patients for the day and I can click through radiographs”—Dental assistant

“I like the efficiency it provides as I go between rooms so that I can more quickly and confidently interpret radiographs.”—Dentist

“Visual aid, ability to toggle on/off visual aid.”—Dentist

Several providers expressed their appreciation for having the AI software to further validate the providers’ initial findings. Additional strengths discussed by providers included, being able to pull up all the patient's radiographs in advance, being able to quickly click through radiographs, ability to turn the AI software annotations on and off, improved efficiency of oral exams, and assistance with diagnosis and treatment planning.

### Qualitative results—patient survey write-in responses

Patients provided additional comments in the survey that the highlighting of dental anomalies made it easier for them to understand what the provider was describing.

“The color changes are helpful. When you say there’s a little grey, it’s hard to tell what you're describing.”

“It was very helpful with the color.”

“Enjoyed the detail.”

The importance of the AI software as an educational tool was also mentioned by a caregiver of a person with limited communication skills.

“[They] sometimes can't find words to describe what [they] are feeling. They are a very sensory oriented person.”

## Discussion

Through the evaluation process, we were able to learn about providers’ knowledge, beliefs, and experiences with using the AI software as a diagnostic, treatment planning, and education tool, and understand if there was an increase in patient satisfaction and treatment plan completion after using the AI software to educate patients about radiographic findings. The provider surveys consistently demonstrated that dental therapists used the AI software the most often and had the most confidence in the use of x-rays as a diagnostic tool and in the AI software as a facilitator for accurate identification of carious lesions and periodontal disease. A decrease in overall provider confidence for using x-rays as a tool alone for identifying dental disease and educating patients suggests providers were beginning to value the AI software as a tool for assisting them with identifying nuances on the radiographs that were not readily noted without assistance. Providers also reported using the AI software to help confirm their initial diagnoses.

At the onset of the AI software training, providers thought that the software alone would diagnose oral conditions. It became clear that additional training was needed on the purpose of using the software as a tool to help diagnose dental disease. Additionally, clarification was needed about the role of the AI software to educate patients about their oral condition with AI-generated annotations on radiographs to indicate carious lesions, bone loss, and indicators of periapical disease. Qualitative survey results brought to light that many providers understood this software as appropriate for use with adult patients only, which is not the case. A desire to have a better login process and for mobile site capability was also expressed by providers.

Although most providers agreed that the AI software would improve the efficiency of their daily work, those who were the most skeptical were dental assistants and dental hygienists. These roles often come with the expectation to have all software and patient information ready when the dentist or dental therapist comes in the patient operatory for examinations or procedures. This would rely on the dental hygienists and dental assistants to take the time to log into the record for each patient.

Overall responses from dental assistants suggested skepticism to the AI software helping patients understand their oral condition and the reason behind treatment recommendations. They spoke about AI not being a dentist and having limited confidence with using AI to identify oral disease. Some dental assistants may not feel confident providing patient education depending on experience level, time allowances, and dentist expectations.

In preliminary studies done for the United States Food & Drug Administration (FDA) approval, the OverJet software demonstrated greater than 85% sensitivity and specificity when measuring periodontal indicators of interproximal bone levels in bitewing and periapical radiographs and periapical bone length (27). In terms of caries detection, the overall sensitivity was 72% (74.4% for primary caries, 62.5% for secondary caries) and the specificity was 98.1% (28). FDA clearance was disclosed to Apple Tree staff as part of training materials provided by the software company, with the ability of staff to access additional information related to accuracy on the software website and FDA publications. As providers’ attitudes toward using AI software can be influenced by their perceptions of the platform's accuracy, this accuracy is an important factor in providers being willing to trust and consistently use such a software platform.

A key component of oral health professionals’ role is patient education. Dental hygienists, due to the preventive and therapeutic nature of their role, are specifically trained in improving patients oral health literacy through motivational interviewing (29, 30). Visual enhancements to standard x-rays provided by the AI software helped the dental providers educate the patients about their oral condition and explain why various treatments were recommended. Anecdotally, this education was particularly effective for minimally invasive techniques as dentists were able to show patients incipient forms of dental caries and education them about the opportunity to prevent caries progression through minimally invasive treatment. This education process was supported by patients’ positive response to seeing the AI software and their willingness to follow through with treatment recommendations.

### Limitations

Future surveys to evaluate the AI software outcomes should have clearly defined inclusion and exclusion criteria for provider survey participation. Study participants included those who did not have ample time to use the AI software based on their type of work setting and confusion with the age range in which the AI software could be used. Also, all questions may not be relevant to the dental assistant role since they are generally not expected to read radiographs or provide patient education on treatment rationale. No demographic data were collected from providers as part of the data collection process beyond provider type. Collecting more provider characteristics (age, years in practice, gender) would support better understanding the uptake of the novel technology and target interventions and be able to compare outcomes between responders and non-responders to the survey. Utilization in the software reports were captured by login data, which may have differed for the provider(s) that utilized the software in clinical care. Less than one year of using a novel technology limits the ability to evaluate the gradient of uptake by providers and into the clinical workflow. Finally, the second provider survey was conducted early in the implementation process; a planned follow-up survey six months after implementation will provide more information about providers’ experiences after the initial learning curve.

Apple Tree Dental recognizes that AI will continue to play a formative role in dental practice and has the potential to support a paradigm of optimal intervention dentistry, whereby conditions are detected earlier and monitored or treated with nonsurgical interventions. The potential for a uniform radiograph annotation tool to calibrate provider diagnosis and treatment planning behavior also appeals to an organization with numerous providers at multiple practice locations. The Innovation Teams have identified opportunities to include this AI software in disease phenotyping efforts through health informatics methods with research collaborators.

Several ethical considerations have emerged through Apple Tree's implementation of the AI software. As a nonprofit organization serving communities who experience health disparities, accessibility to a novel technology was made possible through a technology innovation grant; the cost of continued subscription will depend on availability of additional grant funds or documenting a sufficient return on investment with use of the software. Apple Tree is committed to ensuring that patients have access to high quality dental care, which includes emerging technology that improves oral health outcomes through the early detection and treatment of disease and improved diagnostic calibration. The variable uptake of software use by staff is a challenge that must balance provider professional autonomy with evidence-based professional development and calibration of organizational clinical standards.

## Conclusions

Overall, provider and patient perceptions were positive toward the use of AI to annotate radiographs as both a diagnostic aid and patient education tool. Dentists and dental therapists noted that the software helped facilitate their diagnostic process and helped them better educate their patients about their oral health conditions. Dental hygienists and assistants noted that the AI software added time to their workflow in terms of time needed to open the software individually for each patient. Patients found the AI-produced visual aids used by their dental provider to be helpful in understanding their oral health, and all said they planned to follow through on recommended treatment. More research is needed to determine whether use of AI software in clinical dental practice produces changes in treatment recommendations by providers or in patient adherence to these recommendations.

## Data Availability

The raw data supporting the conclusions of this article will be made available by the authors, without undue reservation.

## Appendix

### Appendix A. Radiographs with and without AI Software Annotations

(Courtesy of Overjet. Disclaimer: Some features may not be available in all areas).

### Appendix B. Provider Survey Regarding Use of AI Software (Survey I and II)

#### Survey I

To understand our baseline attitudes, please watch this [1-minute introductory video] for a brief introduction to [the AI software) and respond to the following questions by [date].

Note: The survey responses are anonymous.

1. Please indicate the type of provider you are.
  ◦ dentist, dental hygienist, dental therapist, dental assistant
2. I feel showing digital radiographs to patients helps in terms of treatment acceptance.
  ◦ Strongly agree -> Strongly disagree
3. How confident are you that radiographs help accurately identify caries?
  ◦ Very confident -> Not at all confident
4. How confident are you that radiographs help accurately identify periodontal disease?
  ◦ Very confident -> Not at all confident
5. I expect [the AI software] to improve the accuracy of identifying caries and periodontal disease.
  ◦ Yes/No/I don't know
6. I expect [the AI software] to improve the efficiency of my daily work.
  ◦ Yes/No/I don't know
7. Compared to radiographs alone, I expect [the AI software] to improve patient treatment acceptance.
  ◦ Yes/No/I don't know
8. Do you have any concerns about using [the AI software]?
  ◦ Yes (insert text box for details), No
9. Please indicate any additional comments here.
  ◦ (Insert text box)

#### Survey II

Note: Changes were made to questions 5 and 8 to reflect active participation with the AI.

1. Please indicate the type of provider you are.
  ◦ dentist, dental hygienist, dental therapist, dental assistant
2. I feel showing digital radiographs to patients helps in terms of treatment acceptance.
  ◦ Strongly agree -> Strongly disagree
3. How confident are you that radiographs help accurately identify caries?
  ◦ Very confident -> Not at all confident
4. How confident are you that radiographs help accurately identify periodontal disease?
  ◦ Very confident -> Not at all confident
5. I feel [the AI software] improves the accuracy of identifying caries and periodontal disease.
  ◦ Yes/No/I don't know
6. I expect [the AI software] to improve the efficiency of my daily work.
  ◦ Yes/No/I don't know
7. Compared to radiographs alone, I expect [the AI software] to improve patient treatment acceptance.
  ◦ Yes/No/I don't know
8. When viewing radiographs, what percentage of the time are you using [the AI software]?
  ◦ Yes (insert text box for details), No
9. Please indicate any additional comments here [open-ended text box]

### Appendix C. Patient Survey Regarding Use of AI Software

1. When do you usually visit the dentist? (Choose all that apply to you.)
  a. When I have a problem.
  b. To get my teeth cleaned.
  c. For dental work (for example, to have a filling done).
2. How often do you visit the dentist? (Choose one.)
  a. Less than 1 time per year
  b. 2-4 times per year
  c. 5 or more times per year
3. Has your dental provider gone over your x-rays with you in the past to show you tooth decay or bone loss?
  a. Yes
  b. No
  c. Don't know
4. At your last appointment, did your dental provider go over your x-rays with you to show you tooth decay or bone loss that were highlighted with the Artificial Intelligence software?
  a. Yes [skip logic to question 4]
  b. No [skip logic to question 5]
  c. Don't know [skip logic to question 5]
5. How do you feel these x-ray highlights helped you to understand your oral health?
  a. Very helpful
  b. Helpful
  c. Slightly helpful
  d. Not at all helpful
6. Do you plan to follow up with your treatment recommendations from your last appointment?
  a. Yes
  b. No
  c. Unsure
  d. I was not given any treatment recommendations at my last appointment
7. Please give us any feedback you would like to share about your last dental experience.
Open response: ____________
8. What is your age in years?
  a. 18-26 years
  b. 27-40 years
  c. 41-64 years
  d. 65 or older
9. Which gender best describes you?
  a. Male
  b. Female
  c. Transgender
  d. Nonbinary
  e. I prefer to identify as __________
10. Which race or ethnic group(s) best describe you? (Choose all that apply.)
  a. White
  b. Black
  c. Asian
  d. American Indian
  e. Hispanic
  f. I identify as ___________
11. What is your preferred language? (Edit responses to reflect languages in your clinic)
  a. English
  b. Spanish
  c. Hmong
  d. My preferred language is _________

### Appendix D. Qualitative Responses from Provider Surveys

Many write in responses were related to survey participants being skeptical and cautious about using an AI platform to recognize oral disease. Although there is apprehension about using new technology there was a general sense of interest, curiosity, and flexible thinking towards the AI software.

There is a change in provider attitude with providers expressing feeling worried about the AI software being used as the tool for diagnosis compared to the second survey where providers are understanding the AI software is a tool to aid in diagnosis and patient education. One dental hygienist mentioned how the detail on the AI software could be used for insurance submission for periodontal therapy.

“Easily visualized caries and restorations to show patients.”—Dental assistant

“I use [the AI software] to confirm my initial diagnosis.”—Dentist

“I have really enjoyed using [the AI software] for… scaling and root planing. It helps me identify calculus deposits that may be smaller or harder to identify on radiographs.”—Dental hygienist

Those who did not favor the AI software made comments related to technology issues such as challenges with frequent log-on, challenges with finding patient records, and slow response time with the software.

“It would be helpful if it could compare radiographs to previous ones to see if carious lesions have progressed.”—Dentist

“AI can't always distinguish between radiographic artifacts and caries.”—Dentist

“The biggest hinderance to more frequent [the AI software] use is that …there are too many clicks and scrolls to get to each patient's radiographs, so unless I have extra time, I just use [radiographic software currently in use].”—Dentist

“Extra step logging in on different computers.”—Dentist

“At times it looks like the perio measurements are not completely accurate.”—Dental hygienist

“Since the x-rays don't show up in [the AI software] right away, I often forget to bring them up later in the appointment when the dentist comes for an exam.”—Dental hygienist

“At times it will appear like decay has been spotted but then it has not been accurate.”—Dental therapist

Comments regarding ease of use suggests some providers have a better understanding on how to use the AI software compared with others.

“[The AI software] lines up all the patients for the day and I can click through radiographs”—Dental assistant

“Visual aid, ability to toggle on/off visual aid.”—Dentist

“Most of the time it makes exams faster.”—Dental assistant

“It assists my doctor and patient treatment.”—Dental assistant

“I like the efficiency it provides as I go between rooms so that I can more quickly and confidently interpret radiographs.”—Dentist

When using the AI software, dentists and dental hygienists may be spending more time on patient education, which can be a challenge when managing appointments but better overall for patient care.

“Using [the AI software] has lengthened the exam time with the dentist, and we start to run behind even more than typical with traditional imaging.”—Dental hygienist

### Appendix E. Demographic Characteristics of Patients Completing Survey (*N* = 25).

**Table:**

Demographic characteristic	*N* (%)
Age	
18–26 years	5 (20)
27–40 years	13 (52)
41–64 years	6 (24)
65 + years	1 (4)
Gender	
Female	20 (80)
Male	5 (20)
Race/ethnicity (choose all that apply)	
White	23 (92)
Black	4 (16)
Asian	0 (0)
American Indian	10 (40)
Hispanic	0 (0)
Open-ended option	0 (0)
Dental visit frequency	
Less than once a year	5 (20)
2–4 times per year	20 (80)
5 + times per year	0 (0)
When do you usually visit the dentist? (choose all that apply)	
When I have a problem	24 (96)
To get my teeth cleaned	23 (92)
For recommended dental work (for example, to have a filling done)	23 (92)
